# Letter from Dr. W. H. Elliot to the Baltimore Editor

**Published:** 1850-01

**Authors:** W. H. Elliot


### ARTICLE III.

### *Letter from* Dr. W. H. Elliot *to the Baltimore Editor.*

Montreal, 24*th Oct.*, 1849.

My Dear Doctor:

Having made a slight alteration in Dr. Hullihen's combination of the screw and forceps, I take the liberty of sending you a description of it. I offer it to the profession at the present moment, in consequence of the discussion which has lately taken place with regard to Dr. Hullihen's invention and Dr. Dubs' improvement upon it, in hopes thereby to save my professional friends the mortification of using a patented instrument.

The improvement consists in making the shaft of the screw about three inches in length, and in passing it directly up through the center of the joint, bringing it out between the handles, and in placing a broad ring upon the upper end of the shaft, sufficiently large to receive the index finger. The joint of the instrument must of course be a single one, and the pivot which forms the fulcrum must be large enough to allow the screw shaft to pass through it, and still leave it sufficiently strong to hold the instrument together. The ring must be put over the screw shaft loosely, so as to turn and adjust itself to the position of the finger.

My instrument has the following advantages over Dr. Dubs', viz. *first,* the operator can pull with the screw without first allowing the forceps to slip back, as Dr. Dubs' does, for the catch to fall into a notch. *Second,* the force may all be given with either the forceps or the screw, or divided between them in such proportion as the operator may think best. *Third,* the instrument is simpler in its construction, and therefore less liable to get out of order. And, lastly, *it is not patented.*

An opening through the joint lengthwise, sufficiently large to receive the shaft of the screw, does not weaken it; nor is it necessary to make the joint different in any way from that of the common single jointed incisor forceps; indeed, one of these instruments may easily have the screw put into it, if it have sufficient room between the handles for the ring.

Should you consider this alteration an improvement, you are at liberty to publish it.

Yours, sincerely and truly,

W. H. ELLIOT.

